# Recent advances in the synthesis of TS-1 zeolite

**DOI:** 10.3389/fchem.2022.1080554

**Published:** 2022-11-22

**Authors:** Huimin Luan, Cheng Xu, Qinming Wu, Feng-Shou Xiao

**Affiliations:** Key Lab of Applied Chemistry of Zhejiang Province, Department of Chemistry, College of Chemical and Biological Engineering, Zhejiang University, Hangzhou, China

**Keywords:** zeolite, TS-1, synthesis, raw materials, routes, nanoscale

## Abstract

Heteroatomic zeolites as an important class of zeolites, have been widely applied in industrially catalytic processes due to their unique properties. As one of the most representative heteroatomic zeolites, titanosilicate zeolites have been extensively used in the selective oxidations of organic substrates with H_2_O_2_ such as cyclohexanone ammoximation, epoxidation of alkenes, and phenol hydroxylation. In this review, recent advances in the synthesis of TS-1 zeolite are briefly summarized, including use of low-cost raw materials (organic templates, silicon, and titanium sources), development of new synthesis routes (post-treatment synthesis, dry-gel conversion synthesis, solvent-free synthesis, and microwave-assisted synthesis), and new strategy for enhanced mass transfer in TS-1 crystals (synthesis of hierarchical and nanosized TS-1 zeolite). This review might help researchers to have a deep understanding on the synthesis of TS-1 zeolite and provide a new opportunity for the design and preparation of highly efficient TS-1 catalysts in the future.

## 1 Introduction

Zeolites have been used in many industrial processes as the efficient catalysts due to their uniform micropores, large pore volumes, high surface areas, and excellent stabilities (Corma, 1995; Cundy and Cox, 2003; Meng and Xiao, 2014). In general, zeolite structures are always consisted of TO_4_ tetrahedra by sharing vertices, where the T atoms are major Si, Al, or P atoms (Arends et al., 1997; Li et al., 2014). In many cases, if T atoms become heteroatoms such as Ti, B, Ga, Fe, it is designated as heteroatomic zeolites (Dusselier and Davis, 2018; Přech, 2018).

As one of the most representative heteroatomic zeolites, TS-1 zeolite is formed by replacing silicon atoms in silicalite-1 zeolite with titanium atoms. In 1983, Taramasso et al. from Italy firstly reported the synthesis of TS-1 zeolite (Taramasso et al., 1983). Later, other titanosilicate zeolites were reported successively, mainly including Ti-Beta, Ti-ZSM-11, Ti-MOR, Ti-MWW, Ti-ITQ-7 and so on (Blasco et al., 1998; Corma et al., 2000; Díaz-Cabañas et al., 2000; Wu et al., 2001; Wu and Tatsumi, 2004; Wang et al., 2007). Among them, TS-1 zeolite has paid much attention due to its wide applications, such as cyclohexanone ammoximation, epoxidation of alkenes, phenol hydroxylation and oxidative desulfurization (Perego et al., 2001).

Due to the introduction of Ti species in the zeolite framework, TS-1 zeolite with MFI structure (Du et al., 2017) has obvious advantages such as good acid resistance, good hydrophobicity, and excellent performance for selective catalytic oxidations (Jin et al., 2007; Chen et al., 2011b). As typical examples, cyclohexanone ammoximation and epoxidation of alkenes have been performed in industrial processes, where TS-1 was employed as catalysts and hydrogen peroxide was used as a green oxidant under mild conditions (Gao et al., 2000; Perego et al., 2001; Cheng et al., 2016). In these oxidations, it is usually regarded that the tetra-coordinated Ti species in the framework and TiO_6_ species are the active centers (Wu et al., 2016; Xu et al., 2020).

In order to increase the catalytic performance and reduce the cost, great efforts have been paid for the synthesis of TS-1 zeolite. As a result, it is developed many new strategies and routes for synthesis of TS-1 zeolites. In this review, we briefly summarized recent advances in the synthesis of TS-1 zeolite, including use of low-cost raw materials, development of new synthesis routes, and preparation of hierarchical and nanosized zeolite crystals.

## 2 Use of low-cost raw materials

### 2.1 Organic templates

Tetrapropylammonium hydroxide (TPAOH) is the first organic template for synthesis of TS-1 zeolite. However, the TPAOH is costly. To reduce the cost, the researchers have made great efforts for use of relatively cheap organic templates to replace TPAOH. Using tetrapropylammonium bromide (TPABr) to replace TPAOH with ammonia used as the alkali source was successful to synthesize TS-1 zeolite (Müller and Steck, 1994). However, the size of the obtained product was larger than that of TS-1 zeolite synthesized with TPAOH as the organic template. To overcome this problem, organic amines as alkali sources such as hexamethylenediamine (Tuel, 1996), methylamine (Shibata et al., 1997), ethanolamine (Liu et al., 2016), and ethylamine (Zuo et al., 2011) were introduced in the synthetic systems.

In the aforementioned systems, the question is whether organic amines act as templates or only as alkali sources. Li et al. (1999) confirmed that the organic amines just acted as alkali sources rather than as organic templates when the amount of TPABr in the gel was enough.

Although the direct ability of organic amines for the synthesis of TS-1 zeolite is much weaker than that of TPA^+^, TS-1 zeolite could be synthesized successfully under the synergistic effect of alkali metal cations or seed crystals with some organic amines. For example, Ma et al. (1996) reported a successful synthesis of TS-1 zeolite in the presence of 1, 6-hexandiamine and n-butylamine as well as sodium hydroxide. However, the presence of sodium ions in the system partially hindered the introduction of Ti species into the zeolite framework, leading to the formation of anatase TiO_2_. Later, Zhang et al. (2009) synthesized TS-1 zeolite in the absence of alkali metal ions using hexamethyleneimine (HMI) with the addition of active TS-1 precursor from the conventional TPAOH system.

### 2.2 Silicon and titanium source

**FIGURE 1:**
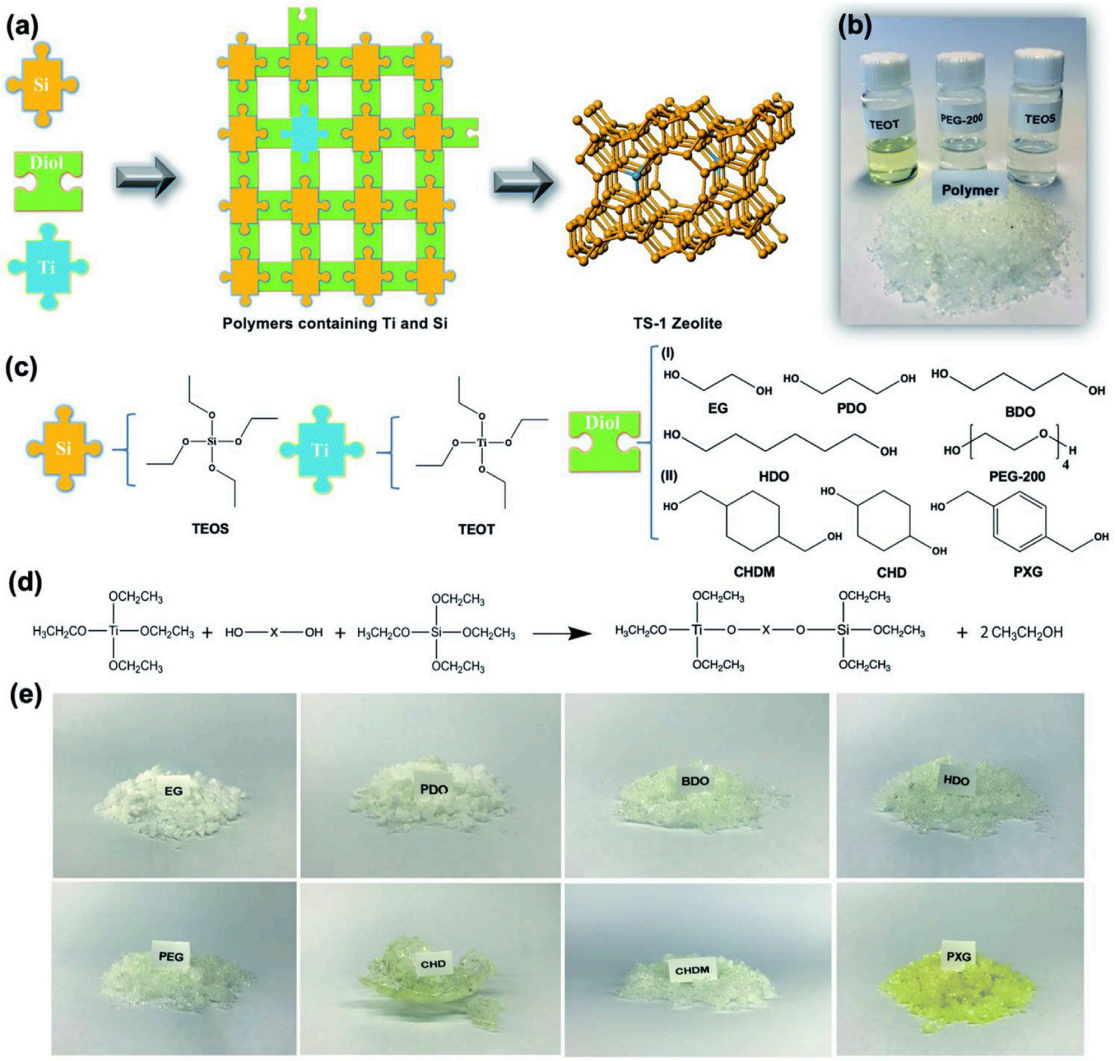
**(A)** Synthesis of TS-1 zeolite from Ti–diol–Si polymers. **(B)** Photograph of the liquid raw materials and solid polymer products. **(C)** Types of alkyl titanates, alkyl silicates and alkyl diols used. **(D)** Transesterification reaction. **(E)** Photograph of the Ti–diol–Si polymers. Reprinted with permission from Xing et al. (2021). Copyright 2021 Royal Society of Chemistry.

It has a great challenge for the synthesis of catalytically active TS-1 zeolite with all titanium species in the framework (Zhang et al., 2016b; Lin et al., 2021), which is strongly related to the selection of silicon and titanium sources in the synthesis. In the beginning, Taramasso et al. (1983) reported that tetraethyl orthosilicate (TEOS) and tetraethyl titanate (TEOT) as silicon and titanium sources were used for the synthesis of TS-1 zeolite. Notably, TEOT hydrolyzed rapidly, partially forming extra-framework titanium species. To solve this problem, Xing et al. (2021) reported the optimized synthesis of TS-1 zeolite from self-made polymer containing titanium and silicon prepared by TEOS and TEOT. Due to the well hydrolysis resistance of Ti-Diol-Si polymer, silicon and titanium sources have suitable matching for the hydrolysis rate in the crystallization process, which is conducive to the formation of high-quality TS-1 zeolite without extra-framework titanium species (Figure 1).

Thangaraj et al. (1992) proposed to replace traditional TEOT with tetrabutyl titanate (TBOT) due to the hydrolysis rate of TBOT matched with TEOS, thus avoiding the formation of extra-framework titanium species. In addition, they also reported the reduction of these extra-framework titanium species in the synthesis by changing the feeding sequence or adding isopropanol.

Compared with organic esters of TBOT and TEOS, inorganic silicon and titanium sources are much cheaper. Thus, the researchers have developed many inorganic titanium and silicon source as the raw materials for the synthesis of TS-1 zeolite. For the inorganic titanium sources, it has been reported titanium fluoride (TiF_4_, Jorda et al., 1997), TiCl_3_ (Gao et al., 1995), TiCl_4_ (Shibata et al., 1997; Zuo et al., 2011), and Ti(SO_4_)_2_ (Zhang et al., 2006). For the inorganic silicon sources, it has been reported colloidal silica (Zuo et al., 2011), fumed silica (Shibata et al., 1997; Zhang et al., 2009), solid silica gel (Zhang et al., 2006). For examples, when TiCl_3_ was used for titanium source (Gao et al., 1995), hydrolysis of TiCl_3_ is relatively slow compared with TBOT, thus avoiding the formation of the extra-framework titanium species. When the TiF_4_ was employed (Shibata et al., 1997; Zuo et al., 2011), it is also avoided the formation of extra-framework titanium species in the TS-1 zeolite because TiF_4_ is more stable than TBOT in the synthesis. Catalytic tests in cyclohexanone ammoximation and hydroxylation of phenol showed that these TS-1 zeolites from inorganic sources were comparable with the TS-1 zeolites from the organic esters. Furthermore, Zhang et al. (2006) reported that solid silica gel and titanium sulfate were used as raw materials to synthesize TS-1 zeolite with good catalytic performance. In this case, a SiO_2_-TiO_2_ precursor with Si-O-Ti bonds was prepared for the hydrothermal synthesis. Table 1 briefly summarized the synthesis of TS-1 zeolites using various raw materials. Obviously, the use of inorganic titanium and silicon as raw materials simplifies the synthetic steps, avoids the generation of anatase, and reduces the cost of TS-1 zeolite.

**TABLE 1:** Overview of the synthesis of TS-1 zeolites using various raw materials.

Entry	Silicon sources	Titanium sources	Template + alkali source	Ref.
1	TEOS	TEOT	TPAOH	Taramasso et al. (1983)
2	TEOS	TBOT	TPAOH	Thangaraj et al. (1992)
3	Ludox AS40	Titanium tetraisopropoxide	TPABr + NH_3_	Müller and Steck (1994)
4	TEOS	TBOT	TPABr + hexamethylenediamine	Tuel (1996)
5	Fumed silica	TiCl_4_	TPABr + methylamine	Shibata et al. (1997)
6	Colloidal silica	TBOT	TPABr + ethanolamine	Liu et al. (2016)
7	Colloidal silica	TiCl_4_	TPABr + ethylamine	Zuo et al. (2011)
8	Fumed silica	TBOT	Hexamethyleneimine	Zhang et al. (2009)
9	TEOS	TiF_4_	TPAOH	Jorda et al. (1997)
10	TEOS	TiCl_3_	TPAOH	Gao et al. (1995)
11	Silica gel	Ti(SO_4_)_2_	TPAOH	Zhang et al. (2006)

## 3 Development of new synthetic routes

### 3.1 Conventional hydrothermal synthesis

Hydrothermal synthesis is a conventional method for synthesis of TS-1 zeolite reported by Taramasso et al. (1983). In general, there are two steps including gelling and crystallization in the hydrothermal synthesis, where anatase is easily produced. Thus, in the process of hydrothermal synthesis, crystallization promoters, protective agents, and/or additives such as isopropyl alcohol, ammonium carbonate, and/or glycine (Thangaraj et al., 1992; Fan et al., 2008; Wang et al., 2016, 2021; Zhang et al., 2018, 2021) would be added to obtain the anatase-free TS-1 zeolite.

**FIGURE 2:**
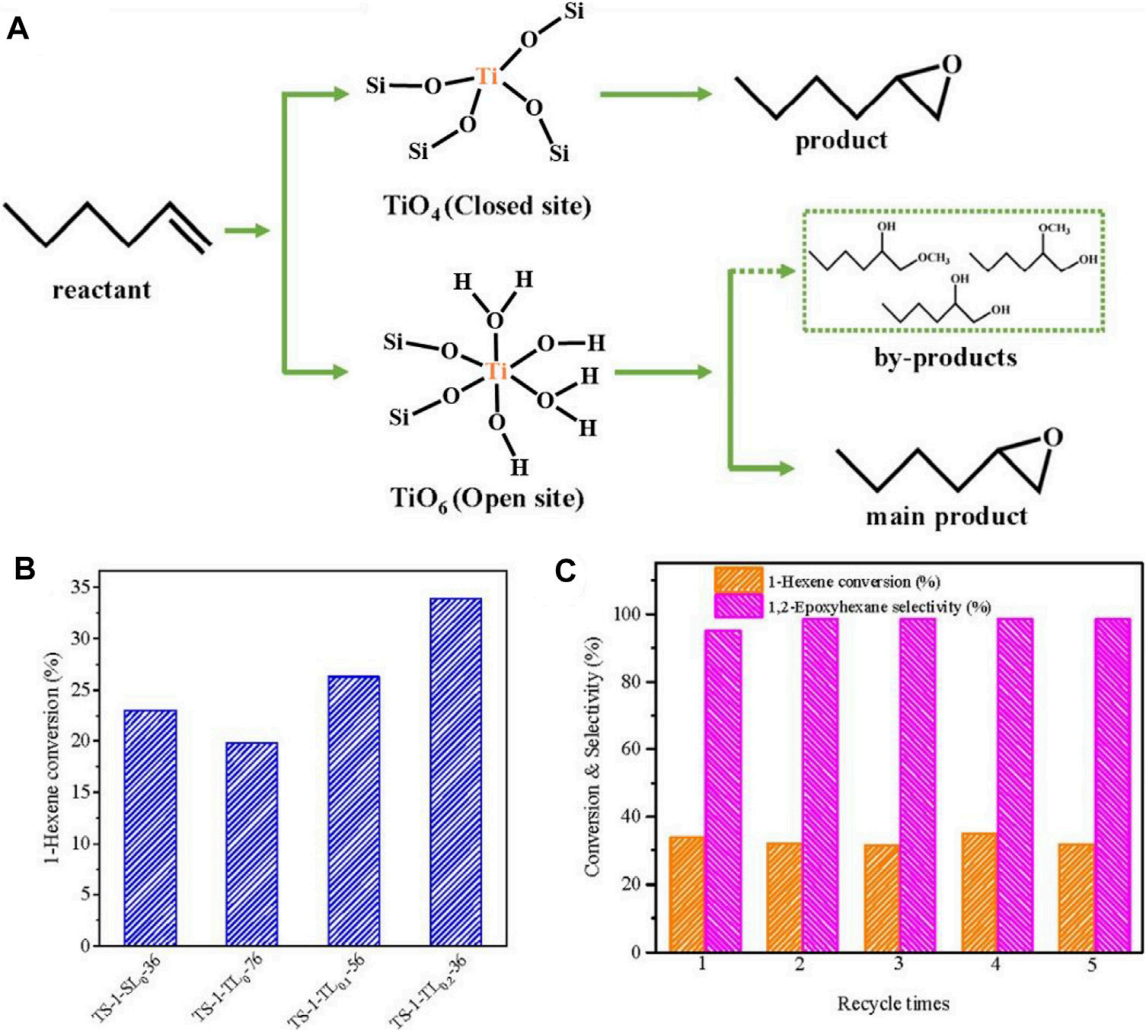
**(A)** Epoxidation routes directed by TiO_4_ (closed sites) and TiO_6_ (open sites) species in the TS-1 zeolites; **(B)** 1-Hexene conversion over the different TS-1 zeolites; **(C)** 1-Hexene conversion and product selectivity of the recycle test using TS-1-TL0.2-36. Reprinted with permission from Wang et al. (2021). Copyright 2021 Elsevier.

Fan et al. (2008) reported that the addition of (NH_4_)_2_CO_3_ could reduce the value of pH and then decrease rate in the crystallization of TS-1 zeolite, which was well matchable with the speed of Ti species entering into the TS-1 framework. As a result, the obtained TS-1 zeolite has high content of Ti species in the framework, giving high catalytic activities in oxidations of a variety of organic substrates. Wang et al. (2021) reported that anatase-free TS-1 zeolite could be synthesized from the help of L-lysine. The presence of L-lysine not only limited the formation of extra-framework titanium species but also ensured efficient incorporation of TiO_6_ (open sites) into the anatase-free TS-1 zeolite. In olefin oxidation of 1-hexene, the TS-1 zeolite from the L-lysine exhibited higher activity than the conventional TS-1, which is owing to the coexistence of TiO_4_ and TiO_6_ species in appropriate proportions in the TS-1 catalyst (Figure 2).

**FIGURE 3:**
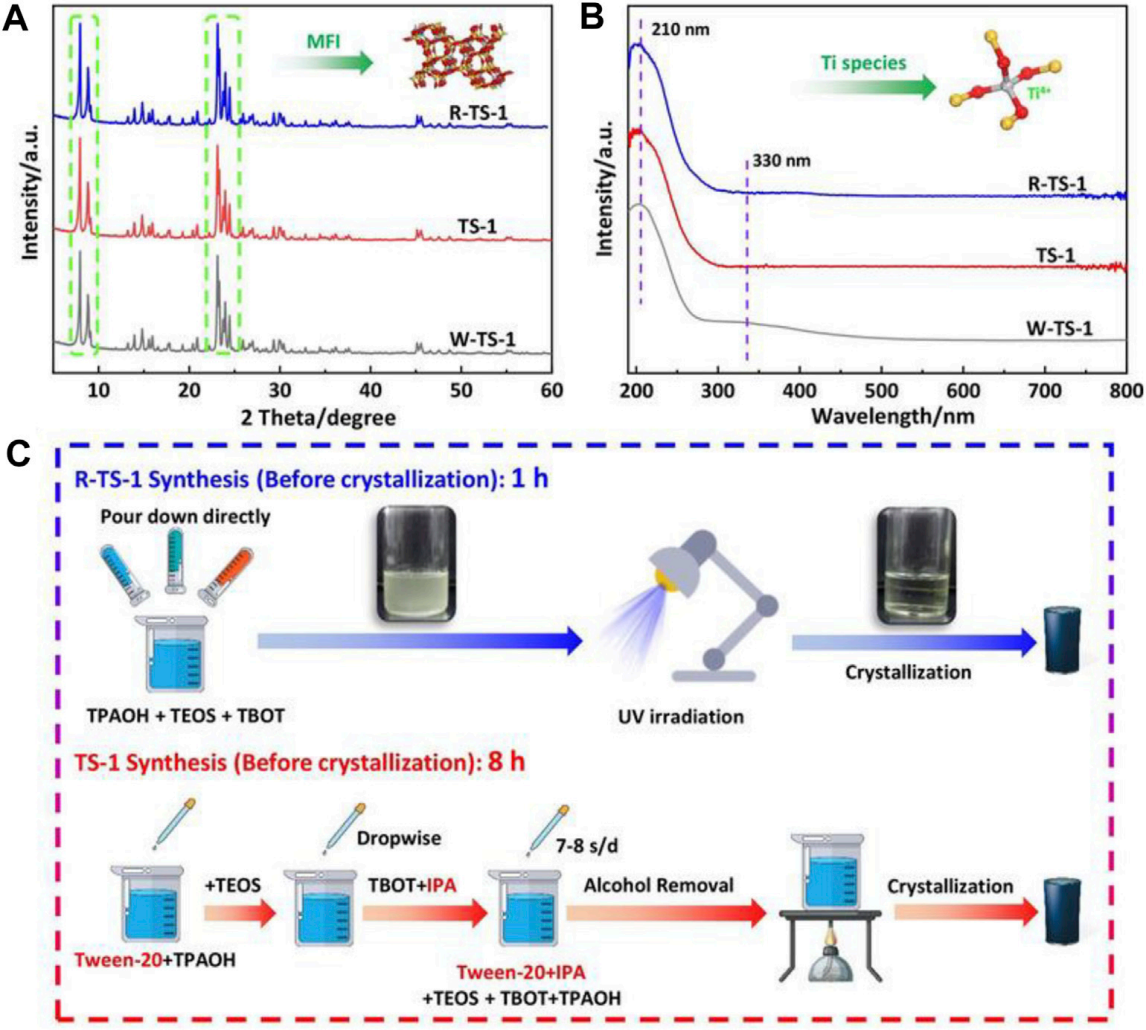
**(A)** XRD patterns and **(B)** UV spectra of R-TS-1 (with UV irradiation and without additives), TS-1 (with additives), and W-TS-1 (without UV irradiation and additives). **(C)** Representation of the synthetic procedures. Reprinted with permission from Lin et al. (2021). Copyright 2020 Wiley-VCH GmbH.

Recently, Lin et al. (2021) reported the reversed-oligomerization with UV irradiation to synthesize TS-1 zeolite by matching the hydrolysis rate of Ti and Si species. Different from the conventional route to avoid the formation of Ti oligomer by slowing down the hydrolysis of Ti precursor, this approach reverses the oligomerization of Ti monomer and accelerates the hydrolysis of Si-alkoxide simultaneously, which is matchable with the hydrolysis rate of Ti species. With the UV irradiation, the hydrolysis time of TEOS reduced from 120 to 60 min and the time of Ti oligomer formation by TBOT was less than 1 min, while the subsequent de-oligomerization of Ti oligomers to Ti monomers was successfully achieved within 60 min (Figure 3).

### 3.2 Post-treatment synthesis

The principle of post-treatment synthesis is to remove B or Al in the structure of ZSM-5 zeolite, generating lattice vacancies, followed by introduction of Ti species into the zeolite framework (Zhang et al., 1999). Generally, the post treatments mainly include gas-solid isomorphous substitution with TiCl_4_ and liquid-solid isomorphous substitution with aqueous solution of (NH_4_)_2_TiF_6_ (Xu et al., 1992; Liu et al., 2006).

Kraushaar and Van Hooff (1988) firstly reported the dealuminated ZSM-5 zeolite with HCl aqueous solution to form lattice vacancies and then reacted with TiCl_4_ to insert titanium species to obtain TS-1 zeolite. The catalytic performance in phenol oxidation for the obtained TS-1 zeolite was similar with that of TS-1 zeolite under hydrothermal conditions (Kraushaar and Van Hooff, 1989).

Yoo et al. (2000) described that the content of Ti in Ti-ZSM-5 zeolite increased with the decreasing of SiO_2_/Al_2_O_3_ ratio when H-ZSM-5 zeolite was a precursor. The structure and surface area of H-ZSM-5 zeolite were not affected in the process of the treatment. Compared with the direct hydrothermal synthesis of TS-1 zeolite, the obtained TS-1 zeolite by the post-treatment synthesis had relatively high conversion and selectivity in cyclohexanone ammoximation.

Liu et al. (2004) reported the effects of B-ZSM-5 zeolite precursors with different molar ratios of SiO_2_/B_2_O_3_ on the incorporation of titanium species into the ZSM-5 framework. With the decrease of SiO_2_/B_2_O_3_ ratio, the more hydroxyl nests could be obtained after HCl treatment, thus more titanium species could be incorporated into the zeolite framework, giving higher catalytic activity in propylene epoxidation.

Post-treatment synthesis of TS-1 zeolite avoids the formation of anatase in the products and the employment of organic titanium as the raw material, which could significantly reduce the cost of TS-1 zeolite. However, this repeatability is relatively poor and synthetic procedures are relatively complex, compared with conventional synthesis of TS-1 zeolite.

### 3.3 Dry gel conversion

In 1990, Xu et al. (1990) firstly reported a dry gel conversion (DGC) to prepare high silica and all silica zeolites. For this method, silica or aluminosilicate gels are mixed with organic templates and then crystallized in the presence of water vapor in a specific reactor. Compared with the hydrothermal synthesis, the dry gel conversion has the advantages of high yield, avoidance of separation, and reduction of wastes (You et al., 2013). Followed this idea, many researchers have devoted to synthesizing TS-1 zeolite using DGC method (Ke et al., 2007; Wang et al., 2013; Wu et al., 2014a; Yue et al., 2014; Zhang et al., 2016a; Du et al., 2019).

Zhang et al. (2016a) showed a one-step DGC method to synthesize shaped TS-1 zeolite by the employment of a small amount of TPAOH. Using this method, the TPAOH/SiO_2_ ratios could be reduced to 0.1, which greatly decreased the cost of TS-1 zeolite. The obtained TS-1 zeolite showed nanosized crystals (50–200 nm). Particularly, it is one step for preparation of the shaped TS-1 zeolite, which is favorable for industrial applications of TS-1 zeolite catalysts.

**FIGURE 4:**
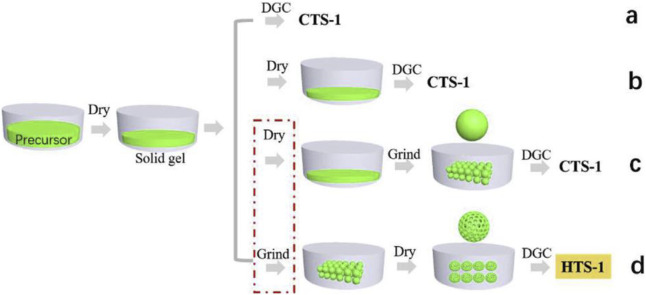
Mechanism of the formation of HTS-1 zeolite. The dry gel with compact structure was converted to conventional TS-1 **(a–c)** and loose compact structure was converted to hierarchical TS-1 **(d)**. Reprinted with permission from Du et al. (2019). Copyright 2018 Elsevier.

Du et al. (2019) synthesized hierarchical TS-1 zeolite by simply adjusting the dry gel preparation process without the addition of mesoporous organic templates using DGC method. The key to this success is to prepare the loosely porous dry gel by grinding, which was helpful for fast diffusion of steam inside the dry gel (Figure 4). Moreover, the surface Ti content of TS-1 zeolite obtained by this method was significantly higher than that of the internal section, contributing to the excellent catalytic performance in the oxidation of bulky sulfur compounds.

### 3.4 Solvent-free synthesis

In recent years, Xiao et al. reported the solvent-free synthesis of zeolites without addition of any solvent (Ren et al., 2012). It has been successfully synthesized pure silica and aluminosilicate zeolites, aluminophosphate and silicoaluminophosphate molecular sieves (Jin et al., 2013, 2014; Wu et al., 2014b; Wu et al., 2019). Compared with conventional synthesis, solvent-free synthesis of zeolites has obvious advantages, such as high zeolite yields, high autoclave utilization, significantly reduced pollutants, reduced energy consumption, simple synthesis processes, and significantly reduced reaction pressures (Meng and Xiao, 2014). Subsequently, it was also successful to synthesize TS-1 zeolite using this method (Zhu et al., 2015; Cui et al., 2017; Fu et al., 2020; Wang et al., 2020; Liu et al., 2022).

**FIGURE 5:**
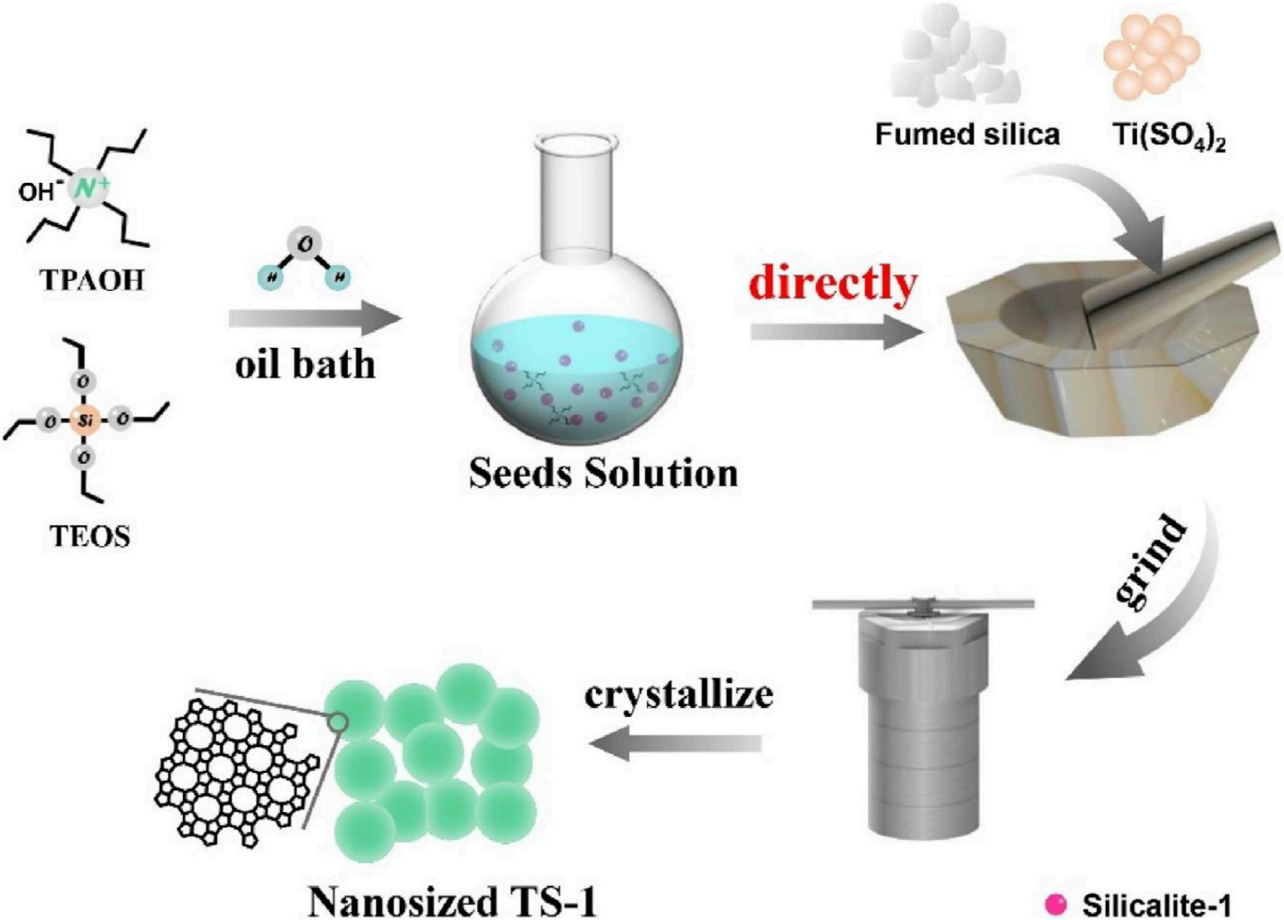
The schematic process of synthesizing anatase-free nanosized TS-1 zeolite under solvent-free conditions. Reprinted with permission from Liu et al. (2022). Copyright 2021 Elsevier.

Zhu et al. (2015) reported the synthesis of TS-1 zeolite using fumed silica, titanium sulfate, TPAOH and zeolite seeds as raw materials under solvent-free conditions. Notably, TS-1 synthesized by this route has almost the same catalytic performance with that of TS-1 zeolite synthesized by conventional hydrothermal method in the catalytic oxidation of hexane. Liu et al. (2022) reported the improved synthesis of TS-1 zeolite from solvent-free synthesis, obtaining the anatase-free nanosized TS-1 zeolite product. It just mixed the untreated seed solution prepared by TPAOH and TEOS, silicon source, and titanium source, then ground and crystallized (Figure 5). By studying the possible mechanism of TS-1 zeolite, it was found that the seed solution is the key factor to obtain nanosized TS-1 zeolite. This method has the advantages of simple operation and high yield, which might open a new opportunity to prepare nanosized TS-1 crystals for industrial applications.

### 3.5 Microwave-assisted synthesis

Microwave-assisted method as a novel route for zeolite synthesis came into the view of researchers in the 1980s and had also been used to synthesize TS-1 zeolite. Prasad et al. (2002) reported TS-1 zeolite could be obtained by microwave-assisted route and the crystallization time of TS-1 (Si/Ti = 33) was as short as 30 min. Moreover, the morphology of TS-1 zeolite could be controlled with microwave irradiation. Jin et al. (2009) showed that the use of microwave led to the formation of the small crystals adhered to each other through the *b*-orientation, forming a stacked TS-1 zeolite. Yang et al. (2006) proved that TS-1 zeolite prepared under microwave irradiation has a high hydrophobicity, leading to the enhanced adsorption capacity of styrene and 1-hexene, thus obtaining the improved catalytic performance of epoxidations.

With the help of microwave irradiation, the modulation of the coordination environments of Ti active sites could also be successful (Xu et al., 2020). TS-1 zeolite with octahedral coordination Ti species (mononuclear TiO_6_) was synthesized by adding zeolite seeds and microwave irradiation. The obtained TS-1 zeolite with mononuclear TiO_6_ feature had good catalytic activity and stability in the epoxidation of 1-hexene.

## 4 The strategies for enhanced mass transfer

Most of the catalytic active site in TS-1 zeolite are located inside of the micropores. In general, the reactants should diffuse into the micropore at first, then access to the active sites. However, the microporous size of the ten-membered ring of TS-1 zeolite is too small to diffuse the reactants and products, which strongly influences the catalytic activities (Du et al., 2016). To solve this issue, it is desirable to synthesize TS-1 zeolite with fast mass transfer (Wang et al., 2022). In the past decades, great advances have been made for synthesis of hierarchical and nanosized TS-1 crystals, effectively eliminating diffusion constraints.

### 4.1 Synthesis of hierarchical TS-1 zeolite

Hierarchical TS-1 zeolite usually has both microporosity and mesoporosity even macroporosity, which not only has fast mass transfer but also reduce the coke formation in the reactions. At present, there are two methods for the synthesis of hierarchical TS-1 zeolite (top-down and bottom-up routes) (Chen et al., 2016). Bottom-up route is to introduce mesopore into TS-1 zeolite by addition of mesoporous templates in the synthesis, including hard templates and soft templates. Top-down route is to introduce mesopore by the extraction of framework composition from post-treatments such as alkali (Valtchev et al., 2011). Many reviews have summarized the synthesis of hierarchical TS-1 zeolite, therefore this part is not discussed in this review (Ren et al., 2017; Yang et al., 2020a; Bai et al., 2021).

### 4.2 Synthesis of nanosized TS-1 crystals

In addition to the hierarchical TS-1 zeolite, nanosized TS-1 crystals are very favorable for fast mass transfer, thus improving the catalytic performances (Wang et al., 2022). At present, the synthesis of nanosized TS-1 crystals mainly includes organotemplate directing, additive assistance, and seed direction.

#### 4.2.1 Organotemplate directing

Organic template not only plays an important role in structural directing but also controls crystalline morphology in the synthesis of zeolites. There are many literatures for synthesis of nanosized TS-1 crystals using unique organic templates (Xu et al., 2019; Shen et al., 2020; Ma et al., 2022). Na et al. (2011) firstly reported that TS-1 zeolite nanosheets with single-unit-cell thickness could be successfully synthesized from quaternary ammonium salt surfactant [C_16_H_33_–N^+^(CH_3_)_2_–C_6_H_12_–N^+^(CH_3_)_2_–C_6_H_13_] as a structure-directing agent. The obtained product has a large external specific surface area (580 m^2^/g) and short diffusion pathway (2 nm along the *b*-axis). 4-coordinated Ti species and more active sites on the external surface of TS-1 zeolite nanosheets resulted in the excellent performance in epoxidation not only for small linear alkenes but also for large alkenes. After reduction of the silanol in the external surface to increase the hydrophobicity of TS-1 zeolite by post-fluoridation, the catalytic activity and epoxidation selectivity of the cycloalkene would be significantly improved.

Li et al. (2020) used the bolaform surfactant [C_6_H_13_–N^+^(CH_3_)_2_–C_6_H_12_–N^+^(CH_3_)_2_–(CH_2_)_12_–O–(*p*-C_6_H_4_)_2_–O–(CH_2_)_12_–N^+^(CH_3_)_2_–C_6_H_12_–N^+^(CH_3_)_2_–C_6_H_13_] as a structure-directing agent to synthesize TS-1 zeolite nanosheets with superior interlayer stability and house-of-cards-like structure. Compared with the traditional TS-1 zeolite and hierarchical TS-1 zeolite synthesized by organosilane surfactant [3-(trimethoxysilyl) propyl] octadecyldimethylammonium chloride (TPOAC), the obtained TS-1 nanosheets exhibited excellent catalytic activity, recovery, and stability for the selective oxidation of bulk cyclic alkenes in liquid phase.

Although TS-1 nanosheets could be synthesized using organic templates, the high cost of organic template limits its practical applications. Further efforts should be done to develop low-cost organic templates for synthesis of TS-1 nanosheets.

#### 4.2.2 Additive-assisted synthesis

Additives such as inorganic and organic agents, polymers, and amino acid, can influence the crystallization of TS-1 zeolite, forming nanosized crystals (Shan et al., 2010; Xu et al., 2012; Shakeri and Dehghanpour, 2019; Yang et al., 2020b; Ji et al., 2021; Li et al., 2021). Compared with designed organic templates, it is economical to use such additives to control the morphology of zeolite crystals.

Li et al. (2021) synthesized TS-1 crystals with the size less than 100 nm by a two-step method using L-lysine as an additive. The introduction of L-lysine inhibited the growth of the crystals, resulting in the formation of nanosized TS-1 crystals. In addition, L-lysine also reduced the pH value of the gel system, which is conducive to incorporate Ti to the TS-1 zeolite framework. Compared with TS-1 zeolite obtained without L-lysine, the conversion of benzene and the yield of phenol increased from 28.9% to 50.2% and 17.1%–30.8%, respectively.

Polyethylene glycol is also a good additive to synthesize nanosized TS-1 crystals with high Ti content in the framework and low content of anatase TiO_2_, resulting in good catalytic performance in hydroxylation of phenol, oxidation of dibenzothiophene, and deep desulfurization of fuels (Shakeri and Dehghanpour, 2019).

**FIGURE 6:**
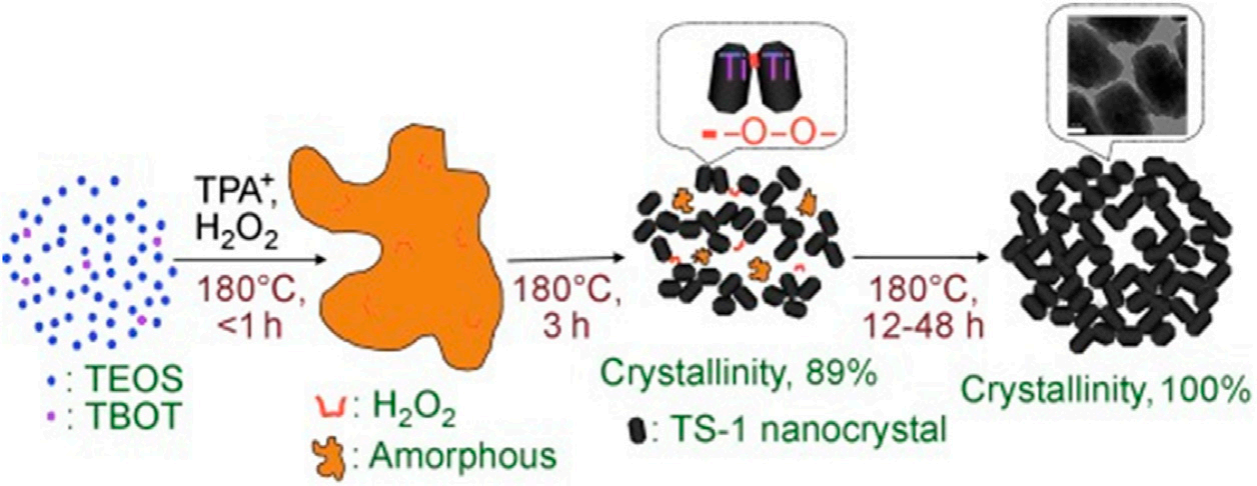
The proposed route for the formation of bulky particles formed by TS-1 zeolite nanocrystals in the presence of H_2_O_2_. Reprinted with permission from Shan et al. (2010). Copyright 2010 Wiley-VCH.

In addition to the organic additives, inorganic additives could be also used for synthesis of nanosized TS-1 crystals. Shan et al. (2010) prepared the TS-1 bulky particles formed by nanocrystals with the addition of H_2_O_2_ and the aggregation of TS-1 nanocrystals was promoted by the strong interaction between Ti species and H_2_O_2_ (Figure 6). Compared with organic additives, the use of inorganic additives has obvious advantage for reduction of the cost.

#### 4.2.3 Seed-directed synthesis

In the process of zeolites synthesis, zeolite seeds could provide crystal nucleus to reduce the crystal size. Nanosized TS-1 crystals has also been prepared by seed-directed synthesis (Chen et al., 2011a; Zuo et al., 2012; Li et al., 2019; Liu et al., 2020).

**FIGURE 7:**
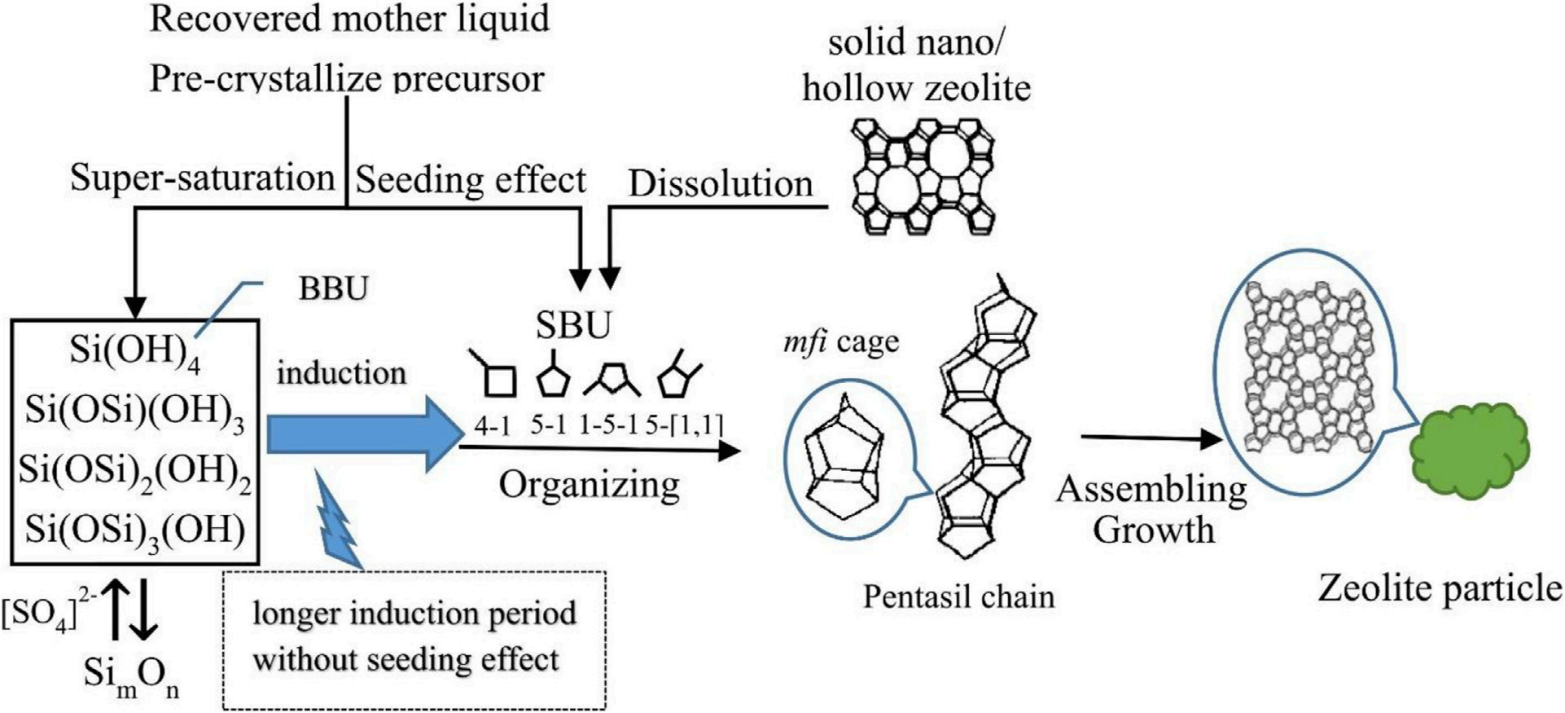
Growth model of the TS-1 zeolite (MFI-type crystal). Reprinted with permission from Liu et al. (2020). Copyright 2019 Elsevier.

Liu et al. (2020) reported that nanosized (50–100 nm) TS-1 zeolite could be efficiently prepared by using the recovered mother liquor within 3–8 h. The growth of TS-1 zeolite can be explained by that the reactive SiO_2_ (especially monosilicate species) polymerized into Si_m_O_n_ species to form the special secondary building units (SBUs) and then these SBUs assembled into the MFI cage, eventually forming the MFI zeolite crystal (Figure 7). The recovered mother liquor not only provides a certain amount of SBU-type Si_m_O_n_ species for fastening the homogenous nucleation but also enhances the supersaturation of active silica species for the formation of nanosized crystals.

Li et al. (2019) synthesized nanoscale TS-1 (360 nm × 190 nm × 640 nm) by seed-directed method in the dry gel system containing tetrapropylammonium bromide (TPABr) and *n*-butylamine. Chen et al. (2011a) obtained hierarchical nanocrystalline TS-1 aggregates with supermicro/mesopores using TPAOH as a single organic template with the assistance of zeolite seeds. The nanocrystalline TS-1 aggregates with the size of 300–500 nm overcame the filtration difficulties in separation, which is helpful for industrial preparation of nanosized TS-1 catalyst at a large scale.

## 5 Conclusion and outlooks

In summary, we simply reviewed recent advances for TS-1 zeolite synthesis. To reduce the TS-1 cost, it is discussed the use of low-cost raw materials including various organic templates, silicon and titanium sources. Furthermore, we described new routes for synthesis of TS-1 zeolite such as post-treatments, dry-gel conversion, solvent-free, and microwave-assisted approaches, which are helpful for reduction of environmentally unfriendly wastes in the synthesis. Finally, it is shown the new strategies for fast mass transfer such as introduction of hierarchical porosity into TS-1 crystals and controllable TS-1 crystals to nanosizes or nanosheets.

Although there are great progresses in the synthesis of TS-1 zeolite, there are still challenges. For examples, industrial preparation of TS-1 zeolite is generally under strong alkaline media, where a large amount of silica species are dissolved in the mother liquor. Therefore, it is strongly desirable to synthesize TS-1 zeolite under near neutral conditions; Currently, it is necessary to use organic templates for the synthesis of TS-1 zeolite, which is costly. Therefore, it is expected to develop an organotemplate-free route for the synthesis of TS-1 zeolite. In view of the wide applications of TS-1 zeolite in the industrial progresses, it should be continuously explored novel strategies for the synthesis of TS-1 zeolite with reduced cost and enhanced catalytic performance.
